# The pathophysiologic significance of lymphocyte subset determination in children with infectious mononucleosis, mycoplasma pneumonia and Henoch–Schönlein purpura

**DOI:** 10.1186/s12887-022-03770-9

**Published:** 2022-12-06

**Authors:** Liang Zhao, Hao Wang, Hua-Xing Wei, Yong Lv

**Affiliations:** 1 Department of Clinical Laboratory, The First Affiliated Hospital of USTC, Division of Life Sciences and Medicine, University of Science and Technology of China, No.9 of Lu Jiang Road, Luyang District, Anhui 230001 Hefei, China; 2 Department of Pediatrics, The First Affiliated Hospital of USTC, Division of Life Sciences and Medicine, University of Science and Technology of China, Anhui 230001 Hefei, China

**Keywords:** Children, Infectious mononucleosis, Pneumonia, Henoch–Schönlein purpura, Cell subsets

## Abstract

**Objective:**

This study aimed to explore lymphocyte subset determinations as an aid to understanding the pathophysiology of infectious mononucleosis (IM), pneumonia due to mycoplasma infection (P-MI) and Henoch–Schönlein purpura in children.

**Methods:**

The peripheral blood lymphocyte subsets of 45 children with IM, 20 children with P-MI, and 31 children with Henoch–Schönlein purpura (HSP), who were treated in the pediatrics department of our hospital from April 2019 to February 2020, were determined by flow cytometry, and the number and percentage of lymphocyte subsets with CD3+, CD3 + CD4+, CD3 + CD8+, CD3 + CD4+/CD3 + CD8+, CD3–CD16 + CD56+, and CD3–CD19 + cells were observed, and the results were compared and analyzed.

**Results:**

(1) The percentages of CD3+, CD3 + CD8 + lymphocyte subsets in children in IM group were significantly higher than those in children with P-MI and HSP, and the percentages of CD3-CD19 + lymphocyte subsets in children in IM group were significantly lower than those in children with P-MI and HSP. The percentages of CD3 + CD4 + lymphocyte subsets in children in the three groups were the lowest in children with IM, and the highest in children with P-MI.The differences in the percentages of CD3+, CD3 + CD4+, CD + CD8+, and CD3-CD19 + lymphocyte subsets among the IM, P-MI, and HSP groups were statistically significant (*P* < 0.01). (2) The results of CD3 + CD4+/CD3 + CD8 + in the three groups were the lowest in children with IM and the highest in children with P-MI. There was a significant difference among the three groups (*P* < 0.01); The ages of the children with IM and P-MI were lower than that of the children with HSP (*p* < 0.01), while there was no difference in the ages of the children with IM and P-MI (*p* > 0.05). (3) The difference in the percentage of CD3–CD16 + CD56 + lymphocyte subsets among the three groups was not statistically significant (*P* > 0.05).

**Conclusion:**

The determination of peripheral blood lymphocyte subsets is of significance for understanding the pathophysiology of IM, mycoplasma pneumonia, and HSP in children.

**Supplementary Information:**

The online version contains supplementary material available at 10.1186/s12887-022-03770-9.

## Background

Infectious mononucleosis (IM) is a proliferative disease of the monocyte–macrophage system that is caused by an Epstein–Barr virus (EBV) infection. Saliva transmission is the main route through which the infection occurs. Pediatric EBV infection is common, and primary EBV infection typically manifests as IM, particularly during adolescence [1, 2]. Globally, EBV is associated with nearly 200,000 cases of cancer and 18,000 multiple sclerosis deaths annually [3, 4], and EBV infection is associated with an increased risk of Hodgkin’s lymphoma and multiple sclerosis [5]. It is unclear why some people develop IM when they are infected with primary EBV but others do not [6]. There is currently a consensus that the vigorous increase in the number of EBV-specific CD8 + cells is a feature of IM, while the change in the proportion of other cell subsets is not well characterized [7].

Mycoplasma pneumonia, an important pathogen that causes human pneumonia, is common in children with respiratory tract infections and has a high incidence rate, and can seriously impact the growth and development of children and their overall health [8]. Most of the clinical manifestations of this disease are subacute. An irritating dry cough and headache are the main clinical manifestations in the initial stage. As the disease progresses, it may cause multiple organ dysfunction and even death [9].

In pediatrics, Henoch–Schönlein purpura (HSP) is a common systemic vasculitis disease involving small blood vessels. Its incidence rate is approximately 1/5,000, which decreases with age; more than 90% of HSP children are younger than 10 years old, and the age of peak incidence is 4–6 years old [10]. The main clinical feature is athrombopenic purpura, which often involves the joints, the gastrointestinal tract, the kidneys, and other organs. At present, the etiology and pathogenesis of HSP are not entirely clear. A study confirmed that the immune imbalance of Th1/Th2 and Th17/Treg cells plays an important role in the pathogenesis of HSP [11].

Infectious mononucleosis, mycoplasma pneumonia infection, and HSP are common diseases that frequently occur among children. Both humoral and cellular immune responses can occur in children with these diseases. The peripheral blood lymphocyte subsets of 45 children with IM, 20 children with pneumonia (mycoplasma infection, [P-MI]), and 31 children with HSP, who were treated in the pediatrics department of our hospital from April 2019 to February 2020, were determined by flow cytometry to explore the disease progression and pathogenesis, thus assisting in the understanding of the conditions noted above.

## Data and methods

### General information

From April 2019 to February 2020, 45 children with IM, 20 children with P-MI, and 31 children with HSP were treated in our hospital. These patients included 51 male and 45 female patients. The age of the patients ranged from 6 months to 13 years old, with an average age of 4.66 ± 2.91 years old. Forty-five children met the inclusion criteria for IM, i.e., any three of the following clinical indicators: (1) fever; (2) pharyngeal tonsillitis; (3) enlargement of the cervical lymph nodes; (4) splenomegaly; (5) liver enlargement; (6) eyelid edema and the diagnosis met the IM criteria. In addition, patients with IM had to meet at least one of the following criteria: (1) a positive serum EBV VCA IgM antibody, (2) a serum EBV VCA IgG that increased > 4 fold from baseline, or (3) a positive blood EBV DNA PCR. The inclusion criteria for mycoplasma pneumonia infection were as follows: (1) all the children in the study group had pulmonary symptoms, e.g., cough, fever, and expectoration, and they all had a mycoplasma pneumonia infection (confirmed by chest CT imaging) and confirmatory serology and/or antigen detection. (2) None of the children received anti-mycoplasma pneumonia or anti-infection treatment. Children with HSP met the diagnostic criteria for this condition formulated by the American College of Rheumatism [12].

### Method

#### Reagents

A CD3/CD45/CD4/CD8 combined reagent, a CD3/CD16 + 56/CD45/CD19 combined reagent, and an erythrocyte lysing solution (a hemolysin) were purchased from Beijing Tongsheng Shidai Biotech Co., Ltd., China.

#### Instrument

A FACSCalibur (Becton, Dickinson and Company, USA) flow cytometry system was used.

#### Method

Fasting venous blood (2 ml) samples were taken from each child in the morning. The blood samples underwent anticoagulation treatment with sodium heparin and were detected within 2 h. Then, 100 µL of anticoagulated samples and 20 µL of mouse anti-human monoclonal antibody were mixed well, left standing for 15 min, added to a 2.0 ml hemolytic solution, incubated at room temperature in the dark for 10 min, and centrifuged at 300 g for 5 min. The supernatant was discarded. The cells were washed twice with phosphate-buffered saline (PBS), added with 1 ml of PBS, and loaded into the instrument. Flow cytometry was conducted according to the instructions specified in the instrument manual, and 15,000 lymphocytes were detected by flow cytometry to determine the percentage of lymphocyte subsets in the peripheral blood.

### Statistical analysis

The data of the experimental results were processed using the SPSS Statistics 17.0 software package. The homogeneity test of variance showed that there was a significant difference in variance (*P* < 0.05). Tamhane analysis was used, and LSD analysis was used for the homogeneity test (*P* > 0.05).Data were expressed as the mean ± standard deviation ($\stackrel{\prime }{x}$ ± SD). Data were compared among multiple groups using an F-test; *P* < 0.05 was considered statistically significant.

## Results

### Variance homogeneity test

A variance homogeneity test of the percentage of lymphocyte subsets with CD3+, CD3 + CD4+/CD3 + CD8+, and CD3–CD19 + cells in children among the IM, P-MI, and HSP groups was carried out, and the results showed that the difference in variance was statistically significant (*P* < 0.05). The variance for CD3 + CD4+, CD3 + CD8+, and CD3–CD16 + CD56 + cells with age was homogenous (*P* > 0.05).

### A comparison of the results among the children in the IM, P-MI, and HSP groups (Table 1)

**Table 1:** Statistical table of counts of lymphocyte subsets in children in the IM, P-MI and HSP groups

Item	Discharge diagnosis	N	Mean value	Standard deviation	Standard error
CD3+(%)	Infectious mononucleosis	45	80.0089^a^	5.70768	0.85085
	Pneumonia (mycoplasma infection)	20	67.5600	8.10292	1.81187
	Henoeh-Schönlein purpura	31	64.0387	8.78786	1.57835
CD3 + CD4+(%)	Infectious mononucleosis	45	14.8978^a^	6.63327	0.98883
	Pneumonia (mycoplasma infection)	20	36.1650^b^	7.50118	1.67731
	Henoeh-Schönlein purpura	31	29.4516^b^	8.33010	1.49613
CD3 + CD8+ (%)	Infectious mononucleosis	45	59.3178^a^	11.19243	1.66847
	Pneumonia (mycoplasma infection)	20	26.2450	6.31477	1.41202
	Henoeh-Schönlein purpura	31	28.2290	7.20977	1.29491
CD3 + CD4+/CD3 + CD8+	Infectious mononucleosis	45	0.2960^a^	0.26402	0.03936
	Pneumonia (mycoplasma infection)	20	1.4755^c^	0.52435	0.11725
	Henoeh-Schönlein purpura	31	1.1165^c^	0.40628	0.07297
CD3-CD16 + CD56+(%)	Infectious mononucleosis	45	10.8156	3.78826	0.56472
	Pneumonia (mycoplasma infection)	20	9.9450	4.93553	1.10362
	Henoeh-Schönlein purpura	31	10.0000	4.62133	0.83001
CD3-CD19+ (%)	Infectious mononucleosis	45	5.0844^a^	4.02673	0.60027
	Pneumonia (mycoplasma infection)	20	18.6900	8.24525	1.84369
	Henoeh-Schönlein purpura	31	23.1742	8.22099	1.47653
Age	Infectious mononucleosis	45	3.8924	2.55345	0.38065
	Pneumonia (mycoplasma infection)	20	3.0330	1.84182	0.41184
	Henoeh-Schönlein purpura	31	6.8387^a^	2.74587	0.49317

^a^The significance level of mean difference is *P* value = 0.000

^b^The significance level of mean difference is *P* value = 0.002

^c^The significance level of mean difference is *P* value = 0.041

1. The percentages of CD3 + and CD3 + CD8 + lymphocytes in the IM group were significantly higher than those in the P-MI and HSP groups, and the differences were statistically significant (*P* < 0.01). There were no significant differences in the percentages of the CD3 + and CD3 + CD8 + lymphocytes between the P-MI and HSP groups (*P* > 0.05).
2. The percentage of CD3–CD19 + lymphocytes in the IM group was significantly lower than in the P-MI and HSP groups, and the difference was statistically significant (*P* < 0.01). The difference in the percentage of CD3–CD19 + lymphocytes between the P-MI and HSP groups was not statistically significant (*P* > 0.05).
3. CD3 + CD4+ (%) results in the children with IM were the lowest and those with P-MI were the highest; these results were statistically significant. The percentages of CD3 + CD4+/CD3+/CD8 + cells in the three groups were the lowest in children with IM and highest in children with P-MI.
4. The difference in the percentage of CD3–CD16 + CD56 + lymphocytes among the IM, P-MI, and HSP groups was not statistically significant (*P* > 0.05).
5. Children with IM and P-MI were statistically significantly younger than the patients with HSP (*P* < 0.01). There was no statistical difference in the ages of the children with IM and P-MI (*P* > 0.05).

## Discussion

Infectious mononucleosis is characterized by fever, angina, lymphadenopathy, eyelid edema, and hepatosplenomegaly. Most Patients with IM have a good prognosis; rare patients develop chronic EBV infection, hemophagocytic lymphohistiocytosis, and malignant tumors. Our data of increased CD8 + and CD56 + cell counts in patients with IM are consistent with what is known about the pathophysiology of IM [13–15], B cell numbers are often the same as those found in healthy controls [14], Why patients with IM had lower B cell numbers than patients with P-IM and HSP is unclear. EBV mainly infects B-cells, causing changes in the surface antigen of these cells, which, in turn, triggers a T-cell (CD3 + cell) defense response. CD3 + CD4 + T-lymphocytes is a type of helper T-lymphocyte, where the intracellular molecules are phosphorylated to activate the signal transduction process and assist in the activation of B-cells, cytotoxic T-cells, and natural killer cells by secreting cytokines, e.g., interleukin (IL)-2 and IL-4. CD3 + CD8 + T-lymphocyte is a cytotoxic type of T-lymphocyte with a killing effect that directly destroys target cells or induces the apoptosis of target cells by secreting perforin interferon-gamma, as well as granzyme [16]. The mean and median ages of our patients with IM, while older than those with P-MI and HSP (3 and 3.8 years; 3 and 3 years; 6.8 and 6 years, respectively) are lower than those seen in the West, where IM is seen mainly in older adolescents and young adults [17].

Mycoplasma pneumonia is a common cause of pneumonia in children. Why these patients had the highest number of CD3 + CD4 + cells of all 3 patients groups is unclear. HSP is a vascular inflammatory disease whose specific immunopathology remains unclear. At present, the excessive activation of autoreactive B-lymphocytes in children with HSP is considered to lead to the excessive production of immunoglobulin A antibody, which is the core mechanism of the disease’s pathogenesis [18]; this is consistent with our findings of the highest number of CD3–CD19 + cells in this condition, as compared with IM and P-IM.

One limitation of our study is the lack of age-matched control groups of healthy children.

## Conclusion

In summary, children with IM, P-MI, and HSP experience immune dysfunction. The dynamic monitoring of peripheral blood lymphocyte subsets can be used to provide insight into the pathophysiology of these three illnesses.

## Supplementary Information

**Additional file 1.**

## Data Availability

The datasets used and/or analysed during the current study available from the corresponding author on reasonable request.

## Abbreviations

IM: Infectious mononucleosis
P-MI: Mycoplasma infection pneumonia
HSP: Henoeh-Schönlein purpura
EBV: Epstein-Barr virus
CD8+: CD8 + T Cell
Th: Helper T
Treg: T-regulatory Cell
BD: Becton,Dickinson and Company
IL: Interleukin
CTL: Cytotoxic T lymphocyte
γ-IFN: γ-interferon
